# Anaesthesia for Paramyotonia Congenita: A Narrative Review

**DOI:** 10.7759/cureus.93393

**Published:** 2025-09-28

**Authors:** Marianne Chee, Suhitharan Thangavelautham, Jing Hui Chen, Harikrishnan Kothandan

**Affiliations:** 1 Anaesthesiology and Critical Care, Singapore General Hospital, Singapore, SGP; 2 Anaesthesiology and Perioperative Medicine, Singapore General Hospital, Singapore, SGP

**Keywords:** anaesthesia for neuromuscular disorders, congenital neuromuscular disorder, eulenburg disease, paramyotonia congenita, pmc

## Abstract

Paramyotonia congenita (PMC) is a rare, non-progressive neuromuscular disorder characterised by muscle stiffness and delayed relaxation following voluntary contraction or mechanical stimulation. Its rarity means many anaesthetists may encounter it only once, if ever, in their careers. This narrative review synthesises existing literature on anaesthetic management in these patients. We aim to provide anaesthetists with a clearer understanding of PMC, with particular focus on perioperative considerations, choice of anaesthetic agents, and reported outcomes. While anaesthetic techniques vary, several recurring management principles emerge, including avoidance of triggers for muscle stiffness, careful temperature control, and readiness to manage potential complications. Although there is no consensus on the optimal anaesthetic approach to PMC, current literature suggests that, with appropriate planning and intraoperative vigilance, safe anaesthesia is achievable.

## Introduction and background

There is a large spectrum of neuromuscular diseases with varying clinical presentations. Many neuromuscular diseases are rare and may not be managed frequently by anaesthetists. Some of these patients will inevitably require surgery and could pose as serious anaesthetic challenges with life-threatening complications, such as malignant hyperthermia.

Paramyotonia congenita (PMC) is a rare non-progressive neuromuscular disease thought to affect fewer than one in 100,000 people [1,2]. Also known as Eulenburg’s disease [3], it was first described in 1886 by von Eulenburg and was considered the first recognised temperature-sensitive condition in humans. It is caused by an over-excitability of muscle fibres after voluntary contraction or mechanical stimulation, resulting in sustained contractions and the inability to relax muscles [1,2]. While the disease can be debilitating, proper management with lifestyle and dietary modifications and medications can allow patients to lead a normal life.

As PMC is very rare, most of the literature on the anaesthetic management for these patients is limited to case reports. There is currently no consensus on the recommended anaesthetic management of these patients. Fortunately, no adverse anaesthetic events have been reported in these case reports. There is, however, a wide range of variation in terms of anaesthetic practices and management strategies.

Our narrative review aims to develop a better understanding of PMC for anaesthetists and discuss the anaesthetic management of previous case reports from the literature.

Pathophysiology

PMC is an autosomal dominant disease caused by point mutations in the SCN4A gene on chromosome 17q [1,2], which encodes for the alpha subunit of the voltage-gated sodium channel in skeletal muscles, known as Nav1.4 [4,5]. Nav1.4 is organised into four homologous domains and six transmembrane segments. Mutations along diﬀerent parts of Nav1.4 are also associated with hyper-, hypo-, and normokalaemic periodic paralysis, cold-aggravated myotonia, potassium aggravated myotonia, and congenital myasthenia syndrome [5]. At least 16 mutations [5] have been found to be associated with PMC. The mutation causes a channelopathy, which results in the unregulated flow of sodium ions into skeletal muscle cells, preventing their repolarisation/relaxation.

Clinical presentation

Myotonia is a key feature of PMC [2]. It is important to understand the pathophysiology of PMC and how the disease manifests clinically. This allows us to differentiate PMC from other myotonic disorders that may mimic it.

PMC presents as episodes of muscle stiﬀness and weakness in the face, neck, and upper limbs of variable duration [6]. Short periods of repeated muscle stimulation can cause stiﬀness, while prolonged overstimulation can cause weakness from muscle fatigue, leading to weakness or flaccid paralysis [2,5,7]. PMC usually presents in early childhood and can be apparent even in infancy [7]. Symptoms can be triggered by exposure to cold or after physical activity, and severity can vary between individuals. Some can manifest as painless myotonia, while others may experience painful myotonia. Patients with the most severe myotonia can sometimes present with shortness of breath or tightness of chest wall muscles. On examination, there is no muscle atrophy, but there is often an increased muscle bulk from hypertrophy. Neurological examination may be normal for patients with PMC.

Investigations

Blood investigations are not diagnostic of PMC, but can help to exclude other diﬀerentials. A renal panel can be performed to evaluate the presence of abnormal potassium or calcium levels. A thyroid function test can also be performed to exclude thyrotoxic periodic paralysis. Creatine kinase (CK) levels can also be measured, as patients with skeletal muscle channelopathy generally have normal to slightly elevated CK levels of up to 1,000 IU/L. CK levels above 1,000 IU/L may suggest an alternative diagnosis [2,5,8].

Standard electromyography and nerve conduction studies may appear normal. However, when combined with a long exercise test, it can help to diagnose periodic paralysis. A short repeat exercise test can show certain patterns of responses that help differentiate between the various non-dystrophic myotonias. PMC patients will demonstrate decreased compound muscle action potential amplitudes during the short repeat exercise test, which is further exacerbated by cooling the muscles. Next-generation genetic sequencing is the gold standard for a definitive diagnosis [2,5,8].

Treatment

As there is no known curative treatment for PMC, treatment is mainly directed at reducing the morbidity of the disease. Conservative measures such as avoidance of foods high in carbohydrates or potassium have been found to be useful. Thus, keeping a food diary can help patients with PMC identify and avoid foods that are possible triggers. Patients should also be cautious to avoid exposure to cold weather and sudden strenuous physical exertion.

Mexiletine, a class 1b antiarrhythmic drug with a high aﬃnity for muscle sodium channels, has some benefit for symptomatic relief. The most common side eﬀects with mexiletine include gastrointestinal eﬀects (e.g., diarrhoea, nausea, and constipation) and neurological eﬀects (e.g., tremors and loss of consciousness). Mexiletine can also cause cardiac side eﬀects such as bradycardia, atrial arrhythmias, first-degree heart block, left bundle branch block, and, rarely, torsades de pointes and new or worsened congestive cardiac failure.

## Review

Literature review

**Figure 1:**
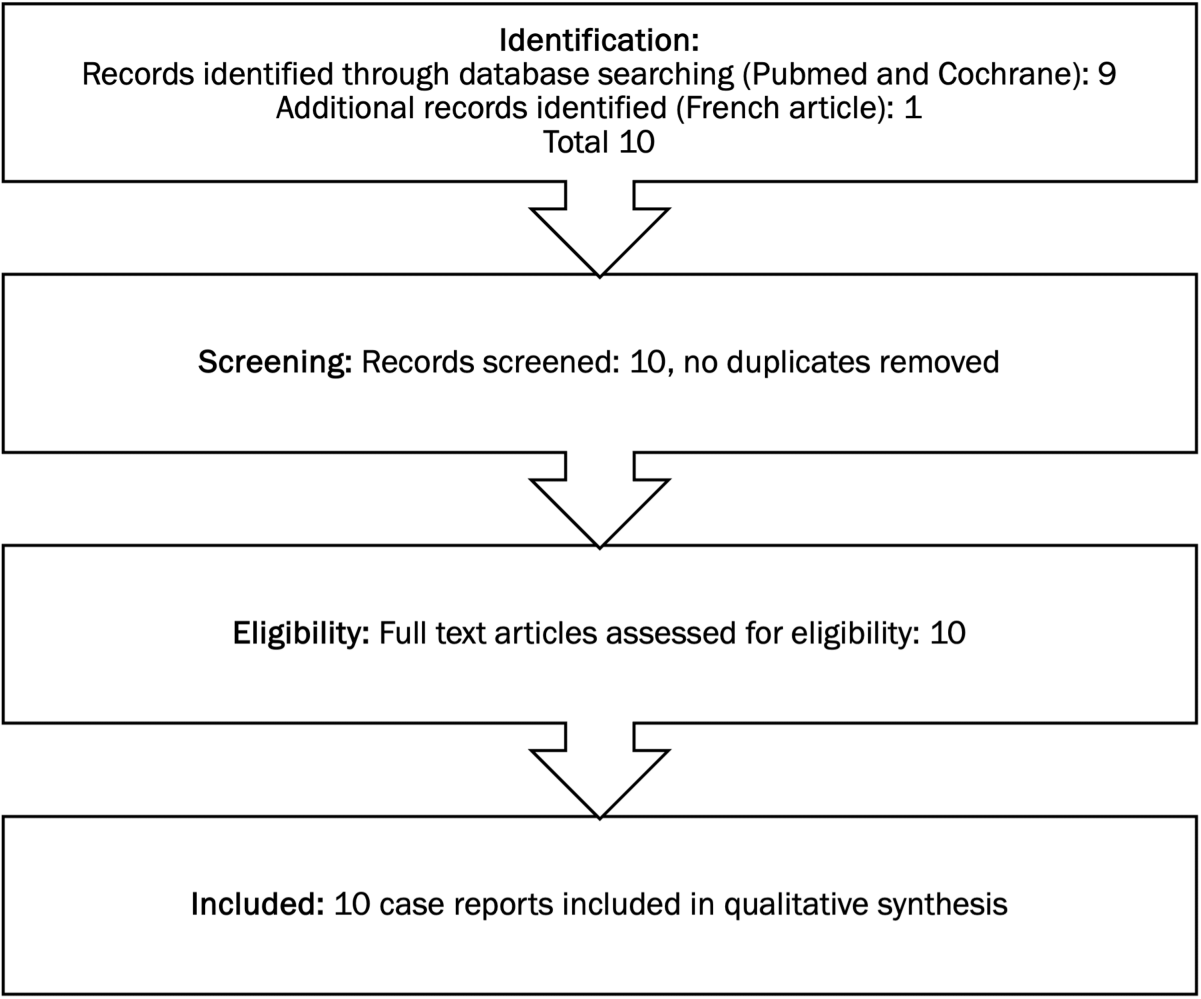
Flowchart of article selection

We conducted a literature search using the PubMed and Cochrane databases. We searched for articles using the keywords "anaesthesia" or "anaesthetic management", "paramyotonia congenita" or "Eulenberg’s disease", and limited the year of publication to 1985. In our initial search, we identified nine case reports that discussed anaesthetic management in patients with PMC, written in the English language. Due to the rarity of the disease, we decided to include another case report that was in French. None of the articles were excluded. Figure 1 shows the flowchart of article selection.

Case Report 1

Reece et al. [9] described a 48-year-old lady who underwent an uneventful general anaesthesia for a mitral valve replacement under moderate hypothermic cardiopulmonary bypass and was extubated five hours postsurgery. She followed a normal postoperative journey and was discharged home on the ninth postoperative day. Anaesthesia was induced intravenously with thiopentone and maintained with fentanyl 300 mcg and droperidol 10 mg. The patient was paralysed with pancuronium and ventilated with a mixture of nitrous oxide and oxygen. No volatile anaesthetics were used to avoid postoperative shivering that could induce myotonia. Additionally, 1 L of Saint Thomas’ solution was used for cardioplegia on top of topical hypothermia. This case report demonstrates that it is safe to undergo cardiopulmonary bypass with moderate hypothermia (28 °C) in patients with paramyotonia congenita. The authors suggest the avoidance of volatile anaesthetics and depolarising muscle relaxants and suggest that a thorough rewarming of patients should be done before weaning off cardiopulmonary bypass.

Case Report 2

Howell et al. [10] described a 31-year-old lady with PMC who had a successful vaginal delivery with a labour epidural inserted. The authors suggest that the use of succinylcholine for rapid sequence induction may lead to myotonic contractures and failure to intubate or open the mouth. Possible alternatives for intubation include an awake intubation or a modified rapid sequence induction using a priming dose or high dose non-depolarising muscle relaxant.

Case Report 3

Grace et al. [11] described a 29-year-old lady with PMC who underwent a caesarean section under spinal anaesthesia. Management included strict temperature management with increasing operating theatre temperature to 24 °C, prewarming of administered intravenous fluids, temperature monitoring, maintenance of body temperature between 36.9 and 37.2 °C, and continuation of postoperative hot-air blanket. There was no intervention on perioperative serum potassium levels, allowing endogenous regulation of electrolytes.

Case Report 4

Binnaz et al. [12] described a two-month-old infant with PMC who underwent a pyloromyotomy and repair of an inguinal hernia. Anaesthesia induction was performed with a minimum alveolar concentration (MAC) of sevoflurane with oxygen. Intubation was performed without muscle relaxants, and anaesthesia was maintained with sevoflurane at 0.5 MAC with a remifentanil infusion at 0.2 mcg/kg/min. The patient recovered full muscular activity postoperatively immediately after discontinuation of sevoflurane and remifentanil. The authors suggest the use of Ringer’s lactate solution without additional potassium supplements for a patient with normal serum electrolytes. They also suggest strict temperature control to avoid hypothermia, as myotonic discharges become more obvious at low temperatures.

Case Report 5

Kaneda et al. [13] described a 70-year-old lady with PMC who underwent a subtotal gastrectomy under general anaesthesia. A T9/T10 epidural was sited with the catheter at 9 cm from the skin. A slow induction was performed with fentanyl and sevoflurane, and endotracheal intubation was performed without muscle relaxants. An epidural bolus was given prior to surgery. Rectal temperature was monitored to ensure that it was maintained between 35.8 and 36.4 °C. Intravenous fluids given were potassium-free. Postoperative analgesia was achieved via a continuous epidural infusion. Succinylcholine was avoided to prevent hyperkalaemia, and non-depolarising muscle relaxants were also not administered as reversal with anticholinesterases could induce myotonic reactions. The authors suggest that local anaesthetic drugs are safe to use for patients with PMC.

Case Report 6

Frossard et al. [14] described a 36-year-old lady, gravidity score of 4 and parity of 2, who underwent successful spontaneous vaginal delivery at 40 weeks with epidural analgesia using ropivacaine infusion. The authors suggest that symptomatic treatment includes limiting exposure to the triggering or aggravating factors. Acetazolamide and sodium channel blockers, such as mexiletine, are effective for reducing the intensity of symptoms.

Case Report 7

Kim et al. [15] described a 33-year-old lady who underwent an open discectomy with a lumbar epidural catheter inserted at the L2/L3 intervertebral space, with a test dose of 3 mL of 1.5% lidocaine with 1:200,000 adrenaline, followed by 20 mL of 0.75% ropivacaine. A propofol infusion was run at a rate of 50-100 mcg/kg/min for sedation. The ambient temperature was raised to 26 °C, and an air warming mattress and pre-warmed fluids were used. The authors suggest that cold sensation should not be used to test for the establishment of the blockade after administering local anaesthetics. Body temperature should also be maintained to avoid shivering. They also suggest that the risk of malignant hyperthermia is not increased as compared with the normal population, as PMC affects the sodium channels rather than the calcium channels.

Case Report 8

Nazuha et al. [3] described a 26-year-old primigravida who had a labour epidural sited after spontaneous rupture of membranes. She then had a top-up of 10 mL of 2% lignocaine for anaesthesia for caesarean section. Temperature management included the use of IV fluid warmers, pre-warmed drapes and disinfection solutions, and pre-warming the operating room to 29.4 °C. The authors suggest the avoidance of electrolyte abnormalities, particularly potassium.

Case Report 9

Brooks et al. [7] described that SCN4A mutations have been identified in infants who present with severe neonatal episodic laryngospasm; however, there is uncertainty as to whether it is an early presentation of myotonia or a separate disease entity. Sodium channel myotonia should be considered early as a differential diagnosis in infants with neonatal hypotonia and stridor with and without a history of PMC or sodium channel mutations. Although PMC affects primarily skeletal muscle, smooth muscle has been reported to contract with cold exposure and induce premature labour, resulting in neonatal death due to prematurity.

Case Report 10

Siow et al. [16] described a 33-year-old lady with PMC and hypokalaemia periodic paralysis, who presented for an elective induction of labour at term. She had an epidural sited for labour, which was topped up for emergency caesarean section. Volatile anaesthetics were avoided to prevent postoperative shivering. Suxamethonium was avoided to prevent inducing myotonia from its hyperkalemic effect, and neostigmine was avoided to prevent inducing myotonia. Postoperatively, she developed persistent bilateral lower limb weakness, which improved when serum potassium levels increased from 3.5-3.7 mmol/L to 4.0 mmol/L.

Anaesthesia management/goals

Preoperative

A multidisciplinary approach should be adopted in the management of such patients. The neurologist should be involved to evaluate the symptoms and severity of the disease, the treatment regimen, and the patient’s response to treatment. A clear history of triggers, such as cold temperature, potassium-rich food, and exercise, must be obtained. Cardiac side effects of mexiletine should also be screened for, and a cardiology review may be warranted pre-operatively.

The use of loop or thiazide diuretics has been described previously by Ashwood et al., as their kaliuretic effects may prevent hyperkalaemia and avoid inducing myotonia [17]. However, there have also been reports of successful anaesthetic management without preoperative diuretics. In addition, a case of prolonged bilateral lower limb weakness was described by Siow et al. in a patient with PMC who had borderline serum potassium levels of 3.5-3.7 mmol/L [16]. This weakness improved after oral potassium supplementation to achieve a serum potassium of 4.0 mmol/L. Thus, we suggest the cautious use of diuretics, unless it is a chronic medication well tolerated by the patient.

Intraoperative

The safe administration of both general and regional anaesthetic techniques has been described. Lehmann-Horn et al. reported that there was no increased susceptibility of malignant hyperthermia (MH) in patients with PMC [18]. This is because PMC is a sodium channelopathy, whilst MH occurs due to a calcium channelopathy.

Induction agents such as thiopentone and propofol have been used in these patients with no adverse effects [9,15]. The use of sevoflurane has proven successful, as described in the case reports. There are no reports about the use of other volatile anaesthetic agents, such as desflurane and isoflurane, in these patients. The use of halothane was avoided by Reece et al. to avoid inducing postoperative shivering, which could have induced myotonia in these patients [9].

The use of suxamethonium has been contraindicated for various reasons. Firstly, its hyperkalaemic effect could induce myotonia. Secondly, its effect on the neuromuscular junction is also known to produce a severe myotonia, causing myotonic muscle rigidity. This can mimic an MH-type reaction, and masseter spasm may make intubation impossible. Although non-depolarising muscle relaxants may not trigger myotonia, the use of anti-cholinesterase drugs for reversal may trigger myotonia. Thus, rocuronium can be considered, should paralysis be required, as sugammadex can be used for reversal to avoid the use of anti-cholinesterases. However, clinical correlation is still advisable to ensure that there is adequate reversal of neuromuscular blockade. There is insufficient data on whether the use of neuromuscular monitoring has been used in these cases, as it is theoretically possible to induce myotonia with modes such as train of four, double burst stimulation, or tetany.

During the maintenance phase of anaesthesia, aggressive efforts to prevent hypothermia should be employed. The use of low fresh gas flows can also further reduce heat loss to the cold and dry fresh gas flow. If a regional technique such as a central neuraxial block is used, one must not forget about the impaired thermoregulation due to the vasodilatory effects of sympathetic blockade. Temperature should be measured, and warm air blankets and fluid warmers should be used to avoid hypothermia. Core temperature should be kept within normal limits to prevent shivering postoperatively, as that is a potential trigger for myotonia.

Postoperative

Care should be made to ensure that the patient remains normothermic by passive or active means of heating. Any shivering should also be treated early with medications such as pethidine or clonidine. Electrolytes, especially potassium, should also be monitored and corrected aggressively.

Discussion

Pregnancy and Myotonia

PMC may affect women of childbearing age. There is currently very limited research on PMC in pregnancy, and most evidence is limited to case reports. Pregnancy has been reported to be one of the triggers of PMC. Brooks et al reported a case of worsening symptoms during the third trimester, although the exact mechanism is unclear [7]. Safe vaginal delivery has been described with the use of epidural analgesia with no reports of myotonic exacerbations during the first or second stage of labour [3,10,14,16]. Several authors have also reported successful caesarean sections performed under central neuraxial techniques [11]. Oxytocics have been used successfully and do not seem to trigger any symptoms.

PMC and Cardiac Surgery

Many cardiac surgical procedures, especially open-heart procedures, require the use of hypothermia during cardiopulmonary bypass for myocardial protection, and a potassium-containing solution for cardioplegic arrest. Cardiac surgical guidelines also emphasise strict glycaemic control of patients, often with the use of insulin. This results in the intracellular shift of potassium ions and could cause hypokalaemia. Off-pump beating heart surgery for procedures such as coronary artery bypass grafting and insertion of a left ventricular assist device could avoid the need for these interventions and hence reduce the risk of triggering symptoms in these patients.

Reece et al. [9] reported a case of a 48-year-old lady with PMC who underwent mitral valve replacement. During cardiopulmonary bypass, she was cooled to 28 °C and was given 1 L of St. Thomas’ solution for cardioplegia. She was slowly rewarmed to 37 °C and weaned off bypass. Postoperatively in the intensive care unit, her core temperature was maintained at 37 °C, and she was successfully extubated five hours after surgery. She remained free of any neuromuscular symptoms throughout her nine days of inpatient stay. This case report has demonstrated that moderate hypothermia with cardiopulmonary bypass can be performed safely without any complications.

## Conclusions

Due to the rare nature of this disease, limited studies can be performed to evaluate the effects of this disease on anaesthesia. Hence, the evidence base is mostly restricted to case reports. Anaesthesia can still be safely performed in these patients, as demonstrated in the various case reports, but does require a detailed understanding of the patient history and triggers of PMC. A detailed preoperative assessment is pertinent to understand the patient's PMC triggers and medical management, as well as to ensure normal electrolyte levels, in particular, serum potassium. Both general and regional anaesthesia techniques have been described safely in these patients. Emphasis should be placed on avoiding the use of depolarizing muscle relaxants and tight temperature control to ensure normothermia. Postoperatively, continual temperature control to prevent postoperative shivering and checking of electrolytes to ensure normal potassium levels.
